# Tandutinib (MLN518) reverses multidrug resistance by inhibiting the efflux activity of the multidrug resistance protein 7 (ABCC10)

**DOI:** 10.3892/or.2013.2362

**Published:** 2013-03-22

**Authors:** WEN DENG, CHUN-LING DAI, JUN-JIANG CHEN, RISHIL J. KATHAWALA, YUE-LI SUN, HAI-FAN CHEN, LI-WU FU, ZHE-SHENG CHEN

**Affiliations:** 1 Department of Pharmaceutical Sciences, College of Pharmacy and Health Sciences, St. John's University, New York, NY 11439, USA; 2 State Key Laboratory for Oncology in South China, Cancer Center, Sun Yat-Sen University, Guangzhou, Guangdong 510060, P.R. China; 3 Guangdong Key Laboratory for Molecular Epidemiology, School of Public Health, Guangdong Pharmaceutical University, Guangzhou, Guangdong, P.R. China; 4 Primary Care Medicine Associates, P.C., Flushing, New York, NY 11354, USA

**Keywords:** multidrug resistance, ATP-binding cassette transporter, multidrug resistance protein 7/ABCC10, FMS-like tyrosine kinase 3, tandutinib

## Abstract

It is well established that ATP-binding cassette (ABC) transporter-mediated multidrug resistance (MDR) is one of the major mechanisms that causes resistance to antineoplastic drugs in cancer cells. ABC transporters can significantly decrease the intracellular concentration of antineoplastic drugs by increasing their efflux, thereby lowering their cytotoxic activity. One of these transporters, the multidrug resistance protein 7 (MRP7/ABCC10), has already been shown to produce resistance to antineoplastic drugs by increasing the efflux of the drugs. In the present study, we investigated whether tandutinib, an FMS-like tyrosine kinase 3 (FLT3) inhibitor, has the potential to reverse MRP7-mediated MDR. Our results revealed that tandutinib significantly enhanced the sensitivity of MRP7-transfected HEK293 cells to the 2 established MRP7 substrates, paclitaxel and vincristine, whereas there was less or no effect on the control vector-transfected HEK293 cells. [^3^H]-paclitaxel accumulation and efflux studies demonstrated that tandutinib increased the intracellular accumulation of [^3^H]-paclitaxel and inhibited the efflux of [^3^H]-paclitaxel from HEK-MRP7 cells. In addition, western blot analysis showed that tandutinib did not significantly affect MRP7 expression. Thus, we conclude that the FLT3 inhibitor tandutinib can reverse MRP7-mediated MDR through inhibition of the drug efflux function and may have potential to be used clinically in combination therapy for cancer patients.

## Introduction

In recent years, the clinical use of surgery, radiation therapy and chemotherapy has reduced the recurrence rates of cancer. However, cellular resistance to chemotherapeutic drugs remains a major obstacle in the successful treatment of cancer (1,2). The efficacy of chemotherapy is limited due to acquired resistance from previous treatment. Consequently, research strategies to circumvent such resistance in cancer cells have become a current focus for the development of novel combination chemotherapy. Both intrinsic and acquired drug resistance can produce multiple changes in various cellular pathways, leading to a decrease in the cytotoxicity, and, thus, a reduction in the efficacy of antineoplastic drugs (3). Therefore, cancer patients that receive chemotherapy can become increasingly insensitive to chemotherapeutic drugs.

One of the primary cellular mechanisms that produces resistance to antineoplastic therapy involves the efflux of drugs from the cancer cells by specific transmembrane transporters or pumps (4). These transporter proteins originate from the superfamily of ATP-binding cassette (ABC) transporters that share common structural and functional properties (5). Previous studies have shown that the majority of the members of the C subfamily of ABC transporters are multidrug resistance proteins (MRPs/ABCCs), which are characterized by cross-resistance to several structurally unrelated drugs (2,4,6,7).

A number of studies suggest that cancer cells that express the ABCC subfamily transporter multidrug resistance protein 7 (MRP7/ABCC10) can develop resistance to various chemotherapeutic drugs. For example, human salivary gland adenocarcinoma (SGA) cells that overexpress *MRP7* mRNA and the MRP7 protein display significant resistance to vincristine (8). MRP7 expression has also been immunohistochemically identified in tumor-bearing mice xenografted with human SGA following treatment with vincristine (8). In addition, E_2_17*β*G, a competitive inhibitor of MRP7 transport, significantly decreased docetaxel accumulation in human SGA cells (8).

The MRP7-overexpressing cells confer resistance to several anticancer drugs including paclitaxel, vincristine and vinblastine (9). Recent studies also reported that MRP7-overexpressing cells confer resistance to nucleoside analogues and epothilone B (10). Furthermore, our laboratory revealed that cepharanthine, a biscoclaurine-derived alkaloid, reversed MRP7-mediated paclitaxel resistance (11).

An important discovery about tyrosine kinase inhibitors (TKIs) was that certain ‘small molecule’ drugs could inhibit TK activity by competing with ATP for binding to the intracellular catalytic domain of receptor TKs, which produced inhibition of various downstream signaling cascades by autophosphorylation (12). Notably, imatinib, nilotinib and dasatinib are inhibitors of the TK breakpoint cluster region-Abelson (*BCR-Abl*) and KIT, a class III receptor TK (13–17). The *BCR-Abl* gene is associated with a dysregulation of TK function, subsequently leading to a malignant transformation in chronic myelogenous leukemia (CML) (18,19). The recognition of the *BCR-Abl* gene and its corresponding protein has led to the development of small-molecule drugs designed to block the activation of *BCR-Abl* TK through competitive binding at the ATP-binding site (18).

In recent years, several experiments determined that TKIs can reverse the resistance of cancer cells to antineoplastic drugs through multiple mechanisms. We and others have reported that some of the TKIs are potent modulators of ABC transporters, including P-glycoprotein (P-gp) and breast cancer resistance protein (BCRP/ABCG2) (20,21). Results from our laboratory suggested that nilotinib significantly reverses P-gp- and BCRP-mediated MDR (22). Our further study found that imatinib and nilotinib can reverse MDR in cancer cells by inhibiting the efflux activity of the MRP7/ABCC10 (23). In addition, we also reported that lapatinib and erlotinib are potent reversal agents for MRP7/ABCC10-mediated MDR (24).

Tandutinib (MLN518/CT53518) is a novel quinazoline-based inhibitor of FMS-like tyrosine kinase 3 (FLT3, a transmembrane receptor in the tyrosine kinase family), platelet-derived growth factor receptor and KIT (25). In the present study, we evaluated the possible interactions of tandutinib with MRP7/ABCC10, with the aim to identify if tandutinib can reverse MRP7/ABCC10-mediated drug resistance. Consequently, it is possible that tandutinib, in combination with other antineoplastic drugs, may be useful in the treatment of cancer that may express MDR proteins, including the ABC transporters.

## Materials and methods

### Materials

Dulbecco's modified Eagle's medium (DMEM), bovine serum and penicillin/streptomycin were purchased from HyClone (Logan, UT, USA). Tandutinib was a product of Selleck Chemicals LLC (Houston, TX, USA). Paclitaxel, fetal bovine serum (FBS), dimethyl sulfoxide (DMSO) and 1-(4,5-dimethylthiazol-2-yl)-3,5-diphenylformazan (MTT), the polyclonal goat antibody against MRP7 (C-19), glyceraldehyde 3-phosphate dehydrogenase (GAPDH), the secondary horseradish peroxidase-labeled anti-goat and anti-mouse IgG were purchased from Sigma-Aldrich Chemical Co. (St. Louis, MO, USA). [^3^H]-paclitaxel (45 mCi/mmol) was purchased from Moravek Biochemicals (Brea, CA, USA). Other routine laboratory reagents were obtained from commercial sources of analytical grade.

### Cell lines and cell culture

HEK293 cells and the MRP7 cDNA were generously provided by Dr Gary Kruh (University of Illinois at Chicago). The HEK293-MRP7-transfected cells and empty vector transfected HEK293-pcDNA3.1 cells were established from HEK293 cells through electroporation (26). Both cell lines were grown as adherent monolayers in flasks with DMEM supplemented with 10% FBS, 2 mM glutamine, 100 U/ml penicillin, and 100 mg/ml streptomycin under standard cell culturing conditions in a humidified incubator containing 5% CO_2_ at 37°C.

### MTT cytotoxicity assay

Prior to the antineoplastic drug sensitivity analysis, we performed the MTT cytotoxicity assay of tandutinib on HEK293-pcDNA3.1 cells and HEK293-MRP7-transfected cells, and the procedure was the same as the following.

Drug sensitivity was analyzed using an MTT colorimetric assay (20). Empty vector-transfected HEK293-pcDNA3.1 cells and HEK293-MRP7-transfected cells were seeded in 96-well plates in triplicate at 5,000 cells/well. Following incubation in DMEM supplemented with 10% FBS at 37°C for 24 h, various concentrations of antineoplastic drugs were added and incubated with the cells continuously for 72 h. For the combination group, a potential inhibitor was added 1 h prior to the addition of an anticancer drug.

Following drug incubation of 72 h, 20 μl MTT (4 mg/ml) was added to each well and the plate was further incubated for 4 h, allowing viable cells to develop from the yellow-colored MTT into dark-blue formazan crystals. Subsequently, the medium was gently removed without agitating the adhesive monolayer of cells, and 100 μl of DMSO was added into each well to dissolve the formazan crystals. The plates were well shaken for 5 min, and an Opsys microplate reader read the absorbance at 570 nm (Dynex Technologies Inc, Chantilly, VA, USA). The degree of resistance was calculated by dividing the IC_50_ for the MDR cells by that of the parental cells, whereas the degree of MDR reversal was calculated by dividing the IC_50_ of the cells with the anticancer drug in the absence of inhibitor by that obtained in the presence of the inhibitor. The concentrations required to inhibit growth by 50% of the control cells were calculated from survival curves using a modified Bliss method (27).

The antineoplastic drugs used in this study included paclitaxel, vincristine and cisplatin at varying concentrations up to a final concentration of 3, 3 and 100 μM, respectively. Tandutinib was used at non-toxic concentrations of 5, 10 and 20 μM and lapatinib at 3 μM to screen against paclitaxel. We subsequently selected their concentrations to determine whether their reversal effects were concentration-dependent to paclitaxel, vincristine and cisplatin.

### Preparation of cell lysates

The cell lines were cultured in DMEM containing 10% FBS at 37°C in the presence of 5% CO_2_. Confluent monolayer cells in T-25 flask were harvested and rinsed twice with cold PBS. The cell extracts were prepared using the Radioimmunoprecipitation assay buffer (1X PBS, 1% Nonidet P-40, 0.5% sodium deoxycholate, 0.1% SDS, 100 mM p-APMSF, 10 mM leupeptin and 10 mM aprotinin) for 30 min on ice with occasional rocking followed by centrifugation at 12,000 rpm at 4°C for 15 min. The supernatant containing total cell lysates was collected and stored at −80°C until use.

### Immunoblotting

Equal amounts of total cell lysates (40 μg) were resolved by 4–12% sodium dodecyl sulfate polyacrylamide gel electrophoresis (SDS-PAGE) and electrophoretically transferred onto nitrocellulose membranes (21). The cell lysates were denatured in a 100°C water beaker for 5 min before loading onto the 4–12% SDS-PAGE. The gel was run in the SDS electrophoresis buffer (25 mM Tris base, 0.192 M glycine, 1% SDS) at 170 V for 2 h. The transfer was performed in a transfer buffer (25 mM Tris base, 0.192 M glycine, pH 8.3) at 80 V for 2 h. The nitrocellulose membrane was then immersed in 5% skim milk to block non-specific binding for 1 h at room temperature. The membrane was then immunoblotted overnight with primary antibodies (polyclonal MRP7 to GAPDH at 1:200 and polyclonal MRP7 at 1:400) at 4°C. The following day, the membrane was washed three times with TBST buffer (0.3% Tris, 0.8% NaCl, 0.02% KCl, 0.05% Tween-20) followed by a 2-h incubation with secondary antibody against GAPDH (ab9483) at 1:2,000. The protein-antibody complex was measured using an enhanced chemiluminescence detection system (Amersham Biosciences, Piscataway, NJ, USA). The membrane was then exposed to the film for development. The conventionally used loading control GAPDH was used to detect equal loading in each lane in the samples prepared from cell lysates.

### Paclitaxel accumulation

Cells in 24-well plates were preincubated with or without tandutinib or lapatinib for 1 h at 37°C, then incubated with 0.1 μM [^3^H]-paclitaxel for 2 h in the presence or absence of the inhibitors (tandutinib or lapatinib) at 37°C. After washing 3 times with ice-cold PBS, the cells were trypsinized and lysed in 10 mM lysis buffer (pH 7.4, containing 1% Triton X-100 and 0.2% SDS). Each sample was placed in scintillation fluid and radioactivity was measured in a Packard TRI-Carb 1900CA liquid scintillation analyzer from Packard Instrument Company, Inc. (Downers Grove, IL, USA).

### Paclitaxel efflux

The HEK293-pcDNA3.1 cells and HEK-transfected cells were seeded in two T-75 flasks and incubated with DMEM supplemented with 10% FBS at 37°C. After the cells were grown to 60–80% confluency, each inhibitor (tandutinib or lapatinib) was added to separate flasks and the cells were incubated for 1 h. The cells were then trypsinized and two aliquots (730,000 cells) from each cell line were suspended in the medium. Subsequently, cells were suspended in the medium containing [^3^H]-paclitaxel at a concentration of 0.1 μM with or without inhibitor for 1 h at 37°C. The incubation medium was replaced by the medium containing only an inhibitor without [^3^H]-paclitaxel. Aliquots (233,000 cells) were collected at various time points (0, 30, 60 and 120 min). The cells were then washed with ice-cold PBS and each sample was placed in scintillation fluid to measure the radioactivity in a Packard Tri-Carb 1900CA liquid scintillation counter from Packard Instrument Inc.

### Statistical analysis

All experiments were repeated at least three times and the differences were determined by two-tailed Student's t-test. When statistical differences between more than 2 groups were analyzed, one-way ANOVA followed by Tukey's multiple comparison test were performed, as indicated. Results are presented as the means ± standard deviations (SD). The statistical significance of differences was determined at P<0.05.

## Results

### Sensitivity of tandutinib on HEK293-pcDNA3.1 and HEK-MRP7-transfected cells

**Figure 1:**
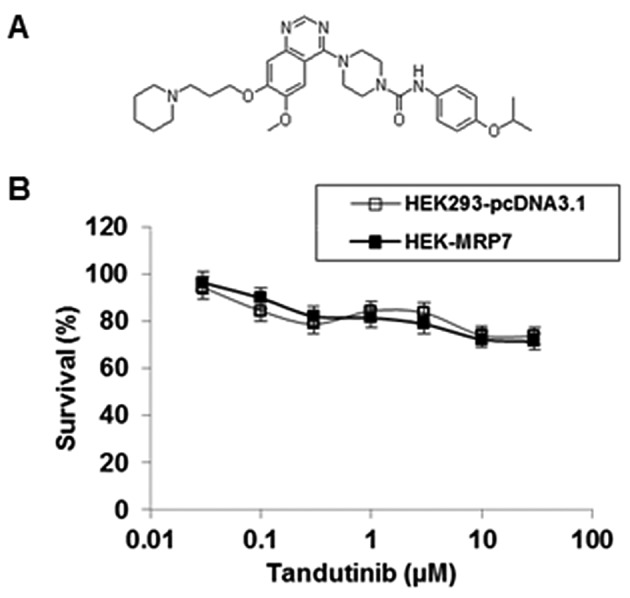
(A) The chemical molecular structure of tandutinib. (B) The sensitivity of tandutinib on HEK293-pcDNA3.1 and HEK-MRP7-transfected cells. Two cell lines, HEK293-pcDNA3.1 and HEK-MRP7, are represented as HEK293 and MRP7, respectively. After seeding and culturing cells for 24 h, different concentrations of tandutinib were added into HEK293-pcDNA3.1 and HEK-MRP7 cells. Three days after culturing, cell survival was determined by MTT assay as described in Materials and methods. Data points are the means ± SD of triplicate determinations. Experiments were performed at least three independent times, and a representative experiment is shown.

To investigate the effect of tandutinib on the MRP7 transporter, we first examined the sensitivity of HEK293-pcDNA3.1 and HEK-MRP7 cells to tandutinib. As shown in Fig. 1B, the IC_50_ values of tandutinib on both HEK293-pcDNA3.1 and HEK-MRP7 cells were >30 μM. Notably, the results of the MTT assay showed that tandutinib did not significantly inhibit the cell growth of these two cell lines at concentrations up to 10 μM (Fig. 1B).

### Effect of tandutinib on the expression of MRP7 in HEK-MRP7 cells

**Figure 2:**
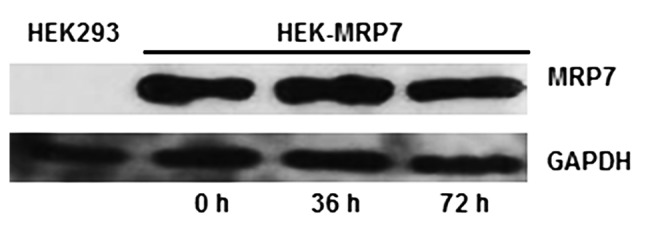
The expression of MRP7 in cells and the effect of tandutinib on the expression of MRP7. Cell lysates were prepared as described in Materials and methods. Equal amounts (50 μg protein) of cell lysates were used for each sample. The effect of 10 μM of tandutinib on the expression levels of MRP7 (upper lanes) in HEK293-pcDNA3.1 and HEK-MRP7-transfected cells for 0, 36 and 72 h, respectively, is shown. The effect of 10 μM of tandutinib on the expression levels of GAPDH (bottom lanes) in HEK293-pcDNA3.1 and HEK-MRP7-transfected cells for 0, 36 and 72 h is shown as loading control. The nitrocellulose membranes were immunoblotted with primary antibody against MRP7 or GAPDH (1:400 dilution), and then incubated with HRP-conjugated secondary antibody at 1:2,000 dilutions at room temperature for 2 h. Each image is a representative example of three replications.

Immunoblot analysis was performed to detect the expression levels of MRP7 protein in the aforementioned lines. MRP7 protein (MW 171 kDa) was expressed in HEK-MRP7 cells, but not in HEK293-pcDNA3.1 cells (Fig. 2). GAPDH, with a molecular weight of 38 kDa, was detected in the HEK293-pcDNA3.1 or HEK-MRP7 cell lines (Fig. 2).

To evaluate the effect of tandutinib on the expression of MRP7, the HEK-MRP7 cells were incubated with 10 μM of tandutinib for 36 and 72 h. The incubation of HEK-MRP7 cells with tandutinib did not significantly alter the expression of the protein levels of MRP7 at different time points (Fig. 2), which is similar to our results for lapatinib (24). This finding suggests that the reversal effect of tandutinib on MRP7-mediated MDR is not due to the regulation of MRP7 expression.

### Analysis of the drug sensitivity of HEK293-pcDNA3.1 and MRP7-transfected HEK293 cells

To determine the drug resistance profile of MRP7, the sensitivity of HEK-MRP7-transfected cells to specific antineoplastic drugs was compared to that of the empty vector-transfected cells, HEK293-pcDNA3.1. The HEK-MRP7 cells exhibited a significantly higher level of resistance to paclitaxel and vincristine (15.94- and 6.25-fold resistance compared to the control cells, respectively) (Table I). These results indicated that the HEK-MRP7 cell line was able to confer resistance to various antineoplastic drugs, which is consistent with our previous reports (11,23).

### Effect of tandutinib on the sensitivity of MRP7-transfected HEK293 cells to anticancer drugs

**Figure 3:**
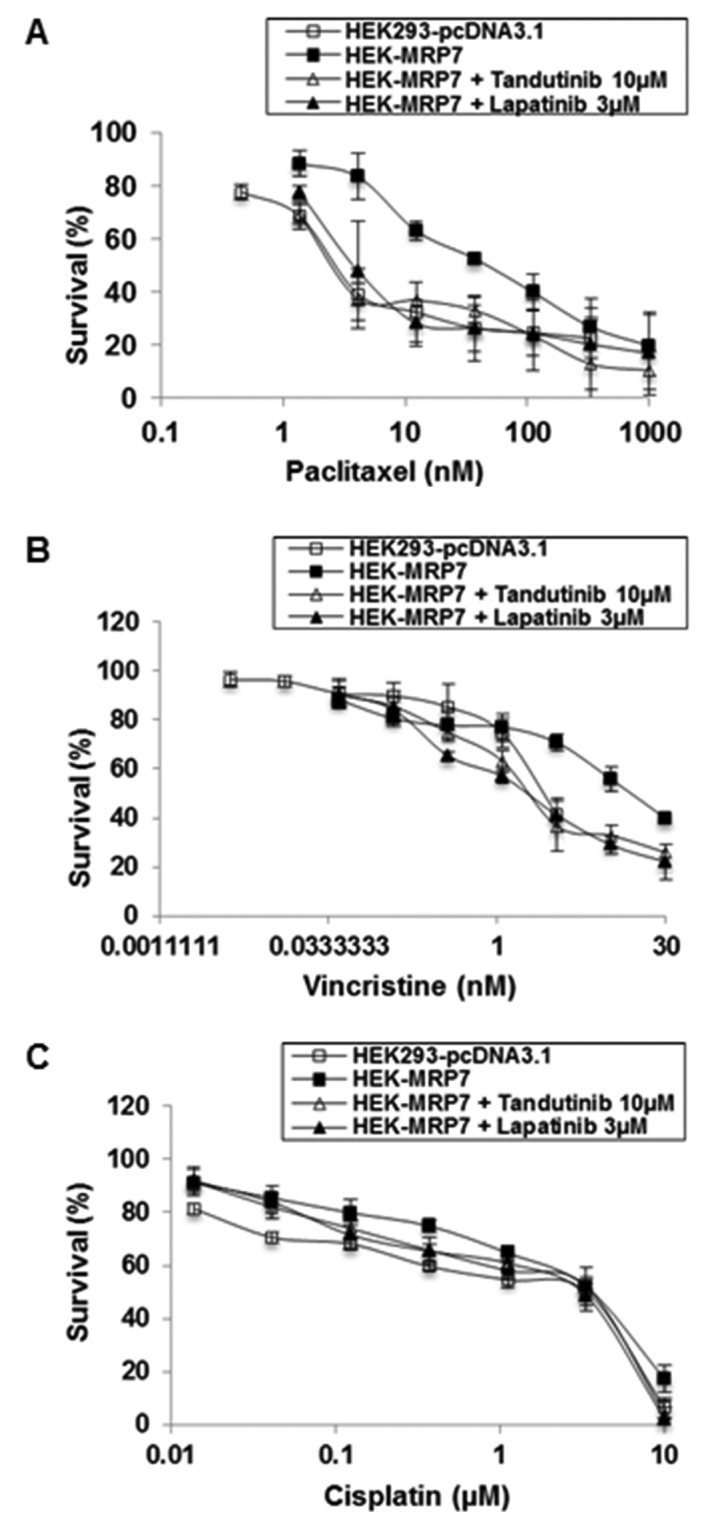
The survival curves of cells at different concentrations of chemotherapeutic drugs in the absence or presence of tandutinib at 10 μM and lapatinib at 3 μM. The two cell lines are HEK293-pcDNA3.1 and HEK-MRP7. After seeding and culturing cells for 24 h, equal amounts of PBS or the reversal agents were added into the HEK293-pcDNA3.1 and HEK-MRP7 cells 1 h before the addition of paclitaxel, vincristine or cisplatin. (A, B and C) The survival curves for the HEK293-pcDNA3.1 (□), HEK-MRP7 (■), HEK-MRP7 + tandutinib 10 μM (△) and HEK-MRP7 + lapatinib 3 μM (▲) at the different concentrations of (A) paclitaxel, (B) vincristine and (C) cisplatin. Cell survival was determined by MTT assay as described in Materials and methods. Data points are the means ± SD of triplicate determinations. Experiments were performed at least three independent times, and a representative experiment is shown.

The preincubation of cells with tandutinib at 10 μM or lapatinib as a positive control, at 3 μM, significantly reversed the resistance of HEK-MRP7 cells to paclitaxel (Table I, Fig. 3A and B). Tandutinib and lapatinib produced a 17.0- and 13.07-fold reversal, respectively, of the resistance to paclitaxel. The IC_50_ of paclitaxel in HEK-MRP7 cells co-cultured with 10 μM of tandutinib was significantly decreased from 56.04±4.09 to 3.3±0.28 nM, which is comparable to that of paclitaxel in HEK293-pcDNA3.1 cells (3.52±0.32 nM). These results suggested that the resistance to paclitaxel was completely reversed when tandutinib was co-incubated with paclitaxel in HEK-MRP7 cells. When HEK293-pcDNA3.1 was co-incubated with tandutinib and paclitaxel, tandutinib, at 5 and 10 μM concentrations, had no significant effect on the sensitivity to paclitaxel. Compared with this sensitivity in HEK293-pcDNA3.1 cells, the sensitivity determined for the MRP7-transfected cells was much greater (Table I, Fig. 3A).

In addition to paclitaxel, we also examined the effect of tandutinib to sensitize cells to another anticancer drug, vincristine. Similar to the findings with paclitaxel, tandutinib (5 and 10 μM) and lapatinib (3 μM) significantly reversed MRP7-mediated vincristine resistance (4.85-, 7.75-fold for tandutinib; and 8.17-fold for lapatinib) in a concentration-dependent manner (Table I and Fig. 3B). We also examined the response of MRP7-transfected cells to a non MRP7 substrate anticancer drug, cisplatin. Our results indicated that tandutinib (5 and 10 μM) did not significantly sensitize the response of HEK293-pcDNA3.1 and HEK-MRP7 cells to cisplatin (Table I and Fig. 3C). This indicates that the response to these drugs is specific for MRP7, as cisplatin is not a substrate for MRP7 and thus would not mediate cisplatin efflux.

In conclusion, tandutinib, similar to lapatinib, significantly reversed MRP7-mediated resistance to paclitaxel and vincristine, but not cisplatin (Table I, Fig. 3).

### Effects of tandutinib on the intracellular accumulation of [^3^H]-paclitaxel

**Figure 4:**
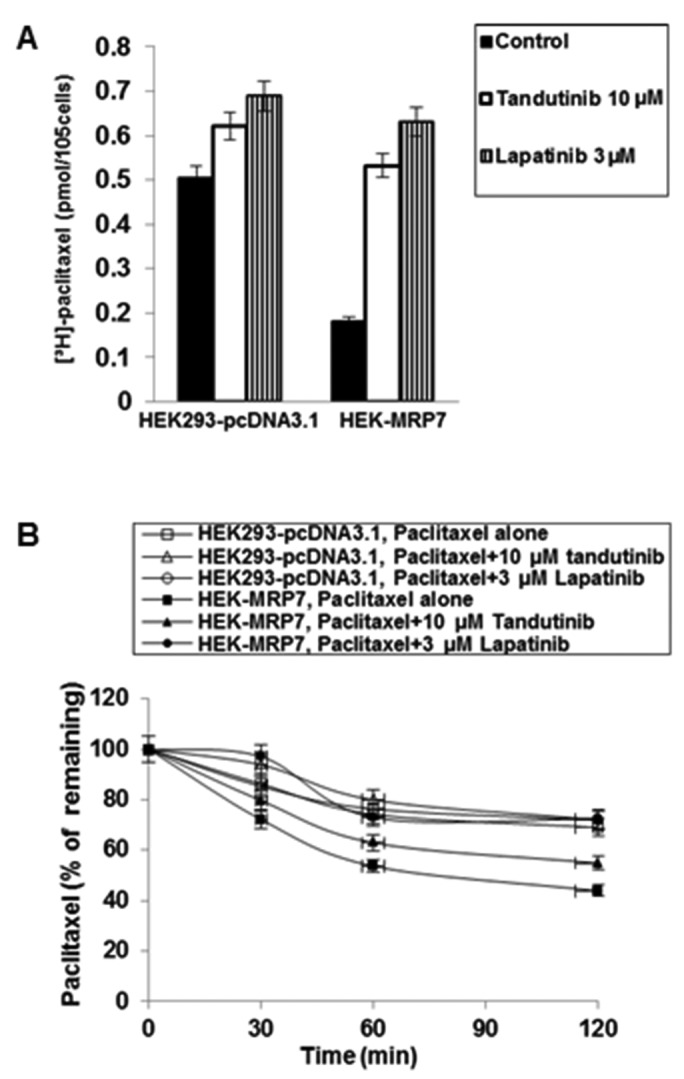
(A) The effects of tandutinib on the accumulation of [^3^H]-paclitaxel. The effects of tandutinib or lapatinib on the accumulation of [^3^H]-paclitaxel in HEK293-pcDNA3.1 and HEK-MRP7 cells are shown. The intracellular paclitaxel accumulations in HEK293-pcDNA3.1 and HEK-MRP7 cells were measured after the incubation with 0.1 μM [^3^H]-paclitaxel. Intracellular accumulation of paclitaxel in HEK293-pcDNA3.1 and HEK-MRP7 cells in the absence of tandutinib and lapatinib is shown on the left. Intracellular accumulation of paclitaxel in the presence of 10 μM of tandutinib in HEK293-pcDNA3.1 and HEK-MRP7 cells is shown in the middle. Intracellular accumulation of paclitaxel in HEK293-pcDNA3.1 and HEK-MRP7 cells in the presence of 3 μM of lapatinib is shown on the right. Each column represents the means (±SD). All experiments were performed in triplicate. ^*^P<0.05, Student's t-test. (B) The effects of tandutinib or lapatinib on the [^3^H]-paclitaxel efflux in HEK293-pcDNA3.1 and HEK-MRP7 cells are shown. The percentage of the paclitaxel released was plotted as a function of time. After 1 h of incubation of the TKIs, [^3^H]-paclitaxel was co-incubated in HEK293-pcDNA3.1 or HEK-MRP7 cells with tandutinib or lapatinib. Cells were washed and re-incubated in the paclitaxel-free medium. At the time points of 0, 30, 60 and 120 min, the cells were collected and the levels of [^3^H]-paclitaxel were determined by scintillation counting. The values at 0 min of drug efflux were set as 1 for comparison to values measured from other time points. Each point represents the means (±SD) of three separate experiments carried out using triplicate samples.

To investigate the potential mechanism by which tandutinib sensitizes MRP7-transfected cells to chemotherapeutic drugs, we examined the effect of tandutinib on the accumulation of [^3^H]-paclitaxel. Intracellular [^3^H]-paclitaxel was measured in MRP7-transfected and empty vector-transfected cells and the results are shown in Fig. 4A. After a 2-h incubation, the intracellular level of [^3^H]-paclitaxel in MRP7-transfected cells was significantly lower than that in the parental HEK293-pcDNA3.1 cells. Tandutinib at 10 μM significantly increased the intracellular level of [^3^H]-paclitaxel similar to the effect of lapatinib at 3 μM in HEK-MRP7 cells.

### Effects of tandutinib on the efflux of [^3^H]-paclitaxel

We previously determined that lapatinib and erlotinib could also reverse MRP7-mediated drug resistance and found that their effects are due to their interaction with the MRP7 protein (24). In the present study, using lapatinib as a comparison agent, we sought to further determine the mechanism by which tandutinib increases intracellular accumulation of [^3^H]-paclitaxel in HEK-MRP7 cells.

It is possible that the increase of intracellular paclitaxel produced by tandutinib is due to: i) a decrease in the efflux of paclitaxel and/or, ii) an increase in the uptake of paclitaxel. HEK-MRP7 cells and HEK293-pcDNA3.1 cells were incubated with paclitaxel and a time course for intracellular paclitaxel remaining was determined (Fig. 4B). HEK-MRP7 cells released a significantly higher percentage of accumulated paclitaxel than HEK293-pcDNA3.1 cells, and the amount of paclitaxel that was effluxed increased with time. When the cells were incubated with tandutinib at 10 μM or lapatinib at 3 μM, they significantly blocked the intracellular [^3^H]-paclitaxel efflux at different time points (0, 30, 60 and 120 min) from HEK-MRP7 cells, but not from HEK293-pcDNA3.1 cells. The accumulation of [^3^H]-paclitaxel at the 0 min time point of efflux was set as 100%, at 30, 60 and 120 min of drug efflux time points, the percentages of the accumulated [^3^H]-paclitaxel that remained in HEK-MRP7 cells in the absence of tandutinib or lapatinib were 72.18, 53.71 and 43.86%, respectively. When HEK-MRP7 cells were incubated with tandutinib, the percentages of intracellular paclitaxel remaining at 30, 60 and 120 min were increased to 79.66, 62.94 and 54.84%, respectively (P<0.05 for the same time point comparison) (Fig. 4B). Lapatinib increased the percentage of [^3^H]-paclitaxel accumulation at 30, 60 and 120 min to 97.08, 72.97 and 72.34%, respectively (P<0.05 for the same time point comparison) (Fig. 4B). Lapatinib was slightly more potent than tandutinib, which is consistent with the results in colorimetric growth assay and [^3^H]-paclitaxel accumulation experiments.

## Discussion

It is well established that MRP7, P-gp and MRP1 are drug efflux pumps responsible for the transport of a variety of antineoplastic drugs from the cells. Consequently, when these pumps are present in the tumor cells concurrently, each of the pumps contributes to the efflux of the drugs and decreases of intracellular drug concentrations. This latter action ultimately leads to lower drug levels that are no longer cytotoxic, leading to the failure of cancer chemotherapy. For instance, nilotinib has been identified as an inhibitor of P-gp and the BCRP efflux pumps (22), and has also been identified as a reversal agent for MRP7-mediated resistance to paclitaxel (23). More recently, we found that nilotinib was also able to revise ABC transporter-mediated MDR in *in vivo* tumor xenograft mouse models (28). These findings suggested for the first time that TKIs may be useful in treating cancer that has become resistant to anticancer drugs as a result of ABC transporter overexpression.

Tandutinib, known as a small-molecule inhibitor of FLT3, is mainly used for acute myelogenous leukemia (AML) and is currently in phase II clinical trials. Its phase I clinical results with tandutinib in patients with AML or high-risk myelodysplastic syndrome showed safety with appropriate pharmacokinetics and pharmacodynamics (25). Previously, Yang *et al*(29) demonstrated that tandutinib was a substrate of P-gp and BCRP. P-gp and BCRP played a role in oral absorption, systemic clearance and brain penetration of tandutinib in the rodents. It has been reported that cepharanthine and nilotinib significantly reversed P-gp-mediated MDR in HEK-MRP7 cells (11) and BCRP-overexpressing cell lines (22), respectively. Our study also found that lapatinib and erlotinib can reverse MRP7-mediated MDR through inhibition of the drug efflux function (24).

In the present study, we determined if tandutinib has the ability to reverse MRP7-mediated drug resistance. We used non-toxic concentrations of tandutinib (10 μM) and lapatinib (3 μM). The transfected HEK293-pcDNA3.1 and HEK-MRP7 cell lines used in our experiments have been used in a previous study from our laboratory (24). We used western blot analysis to detect the expression of MRP7. The cell lines were exposed to the same experimental conditions and procedures, and were cultured with the same antineoplastic drugs for the same incubation time.

We found that tandutinib (10 μM) significantly sensitized MRP7-transfected HEK293 cells to paclitaxel as it markedly decreased the IC_50_ of paclitaxel in MRP7-transfected cells HEK-MRP7, compared to control cells (Table I). In order to extend the findings obtained with paclitaxel, we examined the effect of specific TKIs on the response of MRP7-expressing cells to another antineoplastic drug, vincristine, which is another substrate for MRP7. Our results indicated that tandutinib (10 μM) completely reversed MRP7-mediated vincristine resistance by a factor of 7.75-fold (Table I). Thus, similar to lapatinib, tandutinib significantly attenuates MRP7-mediated resistance to paclitaxel as well as to vincristine. As an additional control, we examined the effect of tandutinib on the response of MRP7-transfected cells to cisplatin, which is not a substrate for MRP7. The results showed that tandutinib (10 μM) did not significantly alter the response of cells to cisplatin (Table I). In addition, our previous study found that P-gp, MRP1 and BCRP were undetectable in both control HEK293-pcDNA3.1 and transfected HEK-MRP7 cells through western blot analysis (24). These results indicated that the HEK-MRP7-transfected cell line specifically expressed MRP7, but not P-gp, MRP1, or BCRP, and the actions of tandutinib in reversing MRP7-mediated resistance are due to a specific effect on the MRP7 pump.

Since the MTT assay results cannot be used as a direct confirmation of MRP7-mediated drug transport, we determined the effect of tandutinib on the accumulation and efflux of [^3^H]-paclitaxel, a known chemotherapeutic substrate of MRP7 transporter, in HEK293-pcDNA3.1 and HEK-MRP7 cells (10). In our experiments, tandutinib significantly increased the intracellular concentration of [^3^H]-paclitaxel, and decreased the intracellular [^3^H]-paclitaxel efflux from the HEK-MRP7 cells but not in the parental HEK293-pcDNA3.1 cells. The reversal effect of tandutinib was similar to that of lapatinib. This suggests that tandutinib modulates MRP7-mediated MDR by increasing intracellular drug accumulation by inhibiting the drug efflux function of MRP7.

In this study, we found that tandutinib significantly decreased resistance to paclitaxel and vincristine in MRP7-overexpressing cells (Table I, Fig. 3) and did not significantly inhibit or induce MRP7 expression (Fig. 2). Collectively, these findings tentatively suggest that tandutinib is capable of reversing MRP7-mediated resistance by inhibiting the function of MRP7.

Previous studies have demonstrated that the systemic exposure achieved at the standard recommended dose of nilotinib is similar to the concentration that reversed drug resistance in *in vitro* cell models (30,31). Previous studies verified that nilotinib is able to revise ABC transporter-mediated MDR both *in vitro* and *in vivo*(22,23,28). Thus, we expect it is also possible that tandutinib is a useful chemosensitizing drug in the clinic for cancer patients if the plasma tandutinib drug concentration can reach 5 μM. Further studies in *in vivo* tumor xenograft studies are required to evaluate the effects of specific FLT3 inhibitors such as tandutinib on the resistance of cancer cells to antineoplastic drugs.

In conclusion, our findings show for the first time that the small-molecule inhibitor of FLT3 tandutinib can effectively reverse MRP7-mediated MDR. The mechanism of MDR modulation by tandutinib is associated with an increase in intracellular drug accumulation by inhibiting drug efflux from MDR cells via MRP7. This suggests that tandutinib could be used to augment the clinical response to conventional chemotherapeutic agents that are substrates of MRP7. Therefore, tandutinib may be a useful modifier of MRP7-mediated MDR in cancer patients.

## Figures and Tables

**Table I:** Effect of tandutinib on reversing MRP7-mediated resistance to paclitaxel, vincristine and cisplatin.

	HEK293-pcDNA3.1		HEK-MRP7	
		
Compounds	IC_50_ ± SD (nM) (RF)	DMF^c^	IC_50_ ± SD (nM) (RF)	DMF^d^
Paclitaxel	3.52±0.32 (1.0)		56.04±4.09 (15.94)	
+Tandutinib 5 μM	3.37±0.32 (0.96)	1.04	11.72±0.74 (3.33)	4.78^d^
+Tandutinib 10 μM	3.42±0.35 (0.97)	1.03	3.30±0.28 (0.94)	17.0^e^
+Lapatinib 3 μM	3.27±0.19 (0.93)	1.08	4.29±0.36 (1.22)	13.07^e^
Vincristine	2.5±0.24 (1.0)^b^		15.6±1.48 (6.25)	
+Tandutinib 5 μM	2.26±0.16 (0.91)	1.10	3.22±0.31 (1.29)	4.85^d^
+Tandutinib 10 μM	1.76±0.15 (0.7)	1.42	2.01±0.21 (0.81)	7.75^d^
+Lapatinib 3 μM	2.02±0.19 (0.81)	1.23	1.91±0.19 (0.77)	8.17^d^
Cisplatin	3428±99 (1.0)		3724±615 (1.09)	
+Tandutinib 10 μM	3515±407 (1.03)	0.98	4335±615 (1.26)	0.86
+Lapatinib 3 μM	3311±367 (0.97)	1.04	3583±430 (1.05)	1.04

Cell survival was determined by MTT assay as described in Materials and methods.

a Data are the means ± SD of at least three independent experiments performed in triplicate.

b Fold-resistance was the value of the IC_50_ value for paclitaxel, vincristine, and cisplatin of HEK293-pcDNA3.1 or HEK-MRP7-transfected cells in the absence or presence of tandutinib.

c Dose-modifying factor (DMF) was the ratio of IC_50_ values without reversal agent compared to the IC_50_ values with reversal agents.

d Significantly different from the control transfected-cells as assayed by the Student's t-test (P<0.05);

e P<0.01.

The experiments were repeated at least three times.

## Abbreviations

MDR: multidrug resistance
ABC: ATP-binding cassette
MRP7: multidrug resistance protein 7
TKI: tyrosine kinase inhibitor
FLT3: FMS-like tyrosine kinase 3

## References

[1] Jemal A, Siegel R, Ward E (2008). Cancer statistics, 2008. CA Cancer J Clin.

[2] Wu CP, Calcagno AM, Ambudkar SV (2008). Reversal of ABC drug transporter-mediated multidrug resistance in cancer cells: evaluation of current strategies. Curr Mol Pharmacol.

[3] Bradbury PA, Middleton MR (2004). DNA repair pathways in drug resistance in melanoma. Anticancer Drugs.

[4] Deeley RG, Westlake C, Cole SPC (2006). Transmembrane transport of endo-and xenobiotics by mammalian ATP-binding cassette multidrug resistance proteins. Physiol Rev.

[5] Borges-Walmsley MI, McKeegan KS, Walmsley AR (2003). Structure and function of efflux pumps that confer resistance to drugs. Biochem J.

[6] Sodani K, Patel A, Kathawala RJ, Chen ZS (2012). Multidrug resistance associated proteins in multidrug resistance. Chin J Cancer.

[7] Chen ZS, Tiwari AK (2011). Multidrug resistance proteins (MRPs/ABCCs) in cancer chemotherapy and genetic diseases. FEBS J.

[8] Naramoto H, Uematsu T, Uchihashi T (2007). Multidrug resistance-associated protein 7 expression is involved in cross-resistance to docetaxel in salivary gland adenocarcinoma cell lines. Int J Oncol.

[9] Hopper-Borge E, Chen ZS, Shchaveleva I, Belinsky MG, Kruh GD (2004). Analysis of the drug resistance profile of multidrug resistance protein 7 (ABCC10): resistance to docetaxel. Cancer Res.

[10] Hopper-Borge E, Xu X, Shen T (2009). Human multidrug resistance protein 7 (ABCC10) is a resistance factor for nucleoside analogues and epithilone B. Cancer Res.

[11] Zhou Y, Hopper-Borge E, Shen T (2009). Cepharanthine is a potent reversal agent for MRP7 (ABCC10)-mediated multidrug resistance. Biochem Pharmacol.

[12] Mendelsohn J, Baselga J (2003). Status of epidermal growth factor receptor antagonists in the biology and treatment of cancer. J Clin Oncol.

[13] Hirota S, Isozaki K, Moriama Y (1998). Gain-of-function mutations of c-KIT in human gastrointestinal stromal tumors. Science.

[14] Shah NP, Nicoll JM, Nagar B (2002). Multiple BCR-ABL kinase domain mutations confer polyclonal resistance to the tyrosine kinase inhibitor imatinib (STI571) in chronic phase and blast crisis chronic myeloid leukemia. Cancer Cell.

[15] Jordanides NE, Jorgensen HG, Holyoake TL, Mountford JC (2006). Functional ABCG2 is overexpressed on primary CML CD34^+^cells and is inhibited by imatinib mesylate. Blood.

[16] Schittenhelm MM, Shiraga S, Schroeder A (2006). Dasatinib (BMS-354825), a dual SRC/ABL kinase inhibitor, inhibits the kinase activity of wild-type, juxtamembrane, and activation loop mutant KIT isoforms associated with human malignancies. Cancer Res.

[17] Hiwase DK, Saunders V, Hewett D (2008). Dasatinib cellular uptake and efflux in chronic myeloid leukemia cells: therapeutic implications. Clin Cancer Res.

[18] Gora-Tybor J, Robak T (2008). Targeted drugs in chronic myeloid leukemia. Curr Med Chem.

[19] Zhang H, Peng C, Hu Y (2012). The Blk pathway functions as a tumor suppressor in chronic myeloid leukemia stem cells. Nat Genet.

[20] Shi Z, Peng XX, Kim IW (2007). Erlotinib (Tarceva, OSI-774) antagonizes ATP-binding cassette subfamily B member 1 and ATP-binding cassette subfamily G member 2-mediated drug resistance. Cancer Res.

[21] Dai C, Tiwari AK, Wu CP (2008). Lapatinib (Tykerb, GW572016) reverses multidrug resistance in cancer cells by inhibiting the activity of ATP-binding cassette subfamily B member 1 and G member 2. Cancer Res.

[22] Tiwari AK, Sodani K, Wang SR (2009). Nilotinib (AMN107, Tasigna) reverses multidrug resistance by inhibiting the activity of the ABCB1/Pgp and ABCG2/BCRP/MXP transporters. Biochem Pharmacol.

[23] Shen T, Kuang YH, Ouyang J (2009). Imatinib and nilotinib reverse multidrug resistance in cancer cells by inhibiting the efflux activity of the MRP7(ABCC10). PLoS One.

[24] Kuang YH, Shen T, Sodani K (2009). Lapatinib and erlotinib are potent reversal agents for MRP7 (ABCC10)-mediated multidrug resistance. Biochem Pharmacol.

[25] DeAngelo DJ, Stone RM, Heaney ML (2006). Phase 1 clinical results with tandutinib (MLN518), a novel FLT3 antagonist, in patients with acute myelogenous leukemia or high-risk myelodysplastic syndrome: safety, pharmacokinetics, and pharmacodynamics. Blood.

[26] Chen ZS, Hopper-Borge E, Belinsky MG, Shchaveleva I, Kotova E, Kruh GD (2003). Characterization of the transport properties of human multidrug resistance protein 7 (MRP7, ABCC10). Mol Pharmacol.

[27] Bliss CI (1935). The calculation of the dose-mortality curve. Ann Appl Biol.

[28] Tiwari AK, Sodani k, Dai CL (2012). Nilotinib potentiates anticancer drug sensitivity in murine ABCB1-, ABCG2-, and ABCC10-multidrug resistance xenograft models. Cancer Lett.

[29] Yang JJ, Milton MN, Yu S (2010). P-glycoprotein and breast cancer resistance protein affect disposition of tandutinib, a tyrosine kinase inhibitor. Drug Metab Lett.

[30] Kantarjian H, Giles F, Wunderle L (2006). Nilotinib in imatinib-resistant CML and Philadelphia chromosome-positive ALL. New Eng J Med.

[31] Tanaka C, Yin OQ, Sethuraman V (2010). Clinical pharmacokinetics of the BCR-ABL tyrosine kinase inhibitor nilotinib. Clin Pharmacol Ther.

